# Prévalence du VIH, de la syphilis et facteurs associés à l'infection au VIH chez les hommes ayant des rapports sexuels avec des hommes et chez les professionnelles du sexe au Togo en 2022

**DOI:** 10.48327/mtsi.v4i3.2024.433

**Published:** 2024-07-08

**Authors:** Panawé KASSANG, Sefako AKAKPO, Kodzo DEKU, Charles LIMAZIE, Nadjombé GBANDI, Anoumou DAGNRA, Palokinam PITCHE

**Affiliations:** 1 Service de dermatologie du CHU Kara, Université de Kara, Togo; 2 Service de dermatologie du CHU Sylvanus Olympio, Université de Lomé, Togo; 3 Conseil national de lutte contre le sida et les infections sexuellement transmissibles (CNLS-IST), Togo; 4 Programme national de lutte contre le sida, les hépatites et les IST, Togo

**Keywords:** Hommes ayant des rapports sexuels avec d'autres hommes, Professionnelles du sexe, VIH, Syphilis, Prévalence, Facteurs associés, Grand Lomé, Tsévié, Kpalimé, Sokodé, Kara, Dapaong, Togo, Afrique subsaharienne, Men who have sex with men (MSM), Female sex workers (FSW), HIV, Syphilis, Prevalence, Associated factors, Grand Lomé, Tsévié, Kpalimé, Sokodé, Kara, Dapaong, Togo, Sub-Saharan Africa

## Abstract

**Introduction:**

Les données de l'infection par le virus de l'immunodéficience humaine (VIH) en Afrique subsaharienne montrent des prévalences élevées dans les populations clés. Les objectifs de cette étude étaient de mesurer la prévalence du VIH, de la syphilis et les facteurs associés à l'infection au VIH chez les hommes ayant des rapports sexuels avec d'autres hommes (HSH) et chez les professionnelles du sexe (PS) du Togo.

**Méthode:**

Nous avons réalisé une étude transversale ciblant les HSH et les PS dans les 6 régions sanitaires du Togo en 2022. La méthode d’échantillonnage basée sur les répondants *(Respondent-Driving Sampling* : RDS) a été utilisée. Les données socio-comportementales, de séroprévalence du VIH et de la syphilis ont été collectées et exprimées en pourcentages et risques pondérés.

**Résultats:**

Au total, 2 110 HSH et 3 221 PS ont été inclus dans notre étude. La moitié des HSH (53,3 %) et des PS (49,7 %) avait un âge compris entre 15 et 24 ans. La prévalence pondérée du VIH était estimée à 8,7 % (IC 95 % : 7,5 % à 9,9 %) chez les HSH et 5,8 % (IC 95 % : 5 % à 6,6 %) chez les PS. Celle de la syphilis était respectivement de 0,6 % (IC 95 % : 0,3 %-0,9 %) et de 0,2 % (IC 95 % : 0 %-0,3 %) chez les HSH et les PS. En analyse multivariée, les HSH ayant : un âge supérieur à 25 ans (OR = 1,71, p=10^-8^); une notion de déchirure de préservatif au cours des douze derniers mois (OR = 1,26, p = 0,001), et plus de deux partenaires sexuels masculins au cours des six derniers mois (OR = 1,96, p=10^-7^), seraient plus à risque de contracter le VIH. Les PS ayant présenté au moins un signe d'infection sexuellement transmissible (IST) au cours des six derniers mois seraient plus à risque de contracter le VIH comparées à celles n'ayant aucun signe d'IST (RC = 1,24, p =10^7^).

**Conclusion:**

Les résultats de notre étude montrent que la prévalence du VIH est élevée au Togo chez les HSH et les PS. Des efforts doivent être redoublés pour avoir un environnement socio-culturel et juridique plus favorable à ces populations.

## Abstract (en)

**Introduction:**

Data on HIV infection in sub-Saharan Africa show high prevalences in key populations. The objectives of this study were to measure the prevalence of HIV, syphilis and factors associated with HIV infection among men who have sex with men (MSM) and female sex workers (FSW) in Togo.

**Method:**

We conducted a cross-sectional study targeting MSM and FSW in the 6 health regions of Togo in 2022. Respondent-Driving Sampling (RDS) was used. Sociobehavioral, HIV and syphilis seroprevalence data were collected and expressed as percentages of balanced risks.

**Results:**

A total of 2 110 MSM and 3 221 FSW were included in our study. Half of the MSM (53.3%) and FSW (49.7%) were aged between 15 and 24 years. The weighted prevalence of HIV was estimated at 8.7% (95% CI: 7.5%–9.9%) among MSM and 5.8% (95% CI: 5%–6.6%) among FSW. Syphilis incidence was 0.6% (95% CI: 0.3%–0.9%) and 0.2% (95% CI: 0%–0.3%), respectively in MSM and FSW. In multivariate analysis, MSM with: an age over 25 years (OR = 1.71; 1,96: p = 10^-8^); a notion of condom tear in the last twelve months (OR = 1.26: p = 0.001), and more than two male sexual partners in the last six months (OR = 1.96: p = 10^-7^), would be at greater risk of contracting HIV. FSW with at least one sign of sexually transmitted infections (STI) in the last six months were at greater risk of contracting HIV than those with no sign of STI (OR = 1.24: p = 10^-7^).

**Conclusion:**

The results of our study show that HIV prevalence is high in Togo among MSM and FSW. Efforts must be redoubled to create a more favorable socio-cultural and legal environment for these populations in order to achieve a greater impact from HIV control interventions.

## Introduction

En 2022, près de 36 millions de personnes vivaient avec le VIH à travers le monde, selon l'ONUSIDA. La prévalence mondiale du VIH chez les adultes (15 à 49 ans) était de 0,7 % alors qu'elle était beaucoup plus élevée dans les populations clés, à savoir les professionnelles du sexe (PS) (2,5 %), les hommes ayant des rapports sexuels avec des hommes (HSH) (7,7 %), les utilisateurs de drogues injectables (UDI) (5 %), les personnes transgenres (10,3 %) et les détenus (1,4 %) [15]. Environ 110 000 (66 000 à 190 000) nouvelles infections aux VIH ont été rapportées la même année en Afrique centrale et de l'Ouest [15]. Des prévalences très élevées ont été rapportées dans certains pays comme le Cameroun (34 %) [17], le Nigéria (34 %) [20] et la Centrafrique (41 %) [13]. Malgré la disponibilité des moyens de prévention et de prise en charge du VIH largement améliorées dans les pays à ressources limitées d'Afrique sub-saharienne, certaines populations clés comme les professionnelles du sexe et les HSH continuent d’être très peu atteintes par ces paquets de services [10], ceci à cause de la stigmatisation et de la discrimination [6]. Les problèmes de stigmatisation constituent un des obstacles à l'atteinte des objectifs de contrôle de l'infection à VIH dans ces populations [8]. Cette situation a amené certains pays comme le Togo à mettre en place une offre de services adaptés pour favoriser l'accessibilité de ces populations aux interventions VIH. Le Togo a adopté depuis 2011 un programme de prévention adapté aux populations clés (HSH, PS, UDI) ainsi qu'un système de surveillance épidémiologique du VIH au travers des enquêtes de surveillance de seconde génération (SSG) dans ces populations clés qui permet de mesurer l'impact des interventions [16]. La présente étude a pour objectif de déterminer les prévalences du VIH et de la syphilis et les facteurs associés au VIH chez les HSH et les PS en 2022 au Togo.

## Matériel et méthodes

### Schéma et période d’étude

Il s'est agi d'une étude observationnelle transversale à visée descriptive et analytique réalisée chez les HSH et PS du 30 août 2022 au 30 septembre 2022 dans les six régions sanitaires du Togo.

### Préparation pré-étude

En amont de l'enquête, une mission exploratoire et de consultation initiale a été réalisée dans les six régions sanitaires du pays pour une meilleure appréhension du contexte de mise en œuvre de l'enquête. Elle a couvert la période allant du 28 juin au 4 juillet 2022. Elle avait pour objectif principal de collecter des données et des informations permettant de faire des derniers ajustements en termes de stratégie opérationnelle de mise en œuvre de l'enquête. En sus, elle a permis de garantir l'adhésion et l'engagement des cibles et des parties prenantes, d’échanger sur les objectifs et la méthode d’échantillonnage d'une part, et de tisser des liens avec la communauté HSH, les PS et les différents partenaires d'autre part.

### Sélection des sites et de la population d’étude

L'enquête exploratoire et de consultation initiale a conduit à se rapprocher des communautés HSH et PS, et à impliquer de manière participative et inclusive leurs membres dans l'identification des sites d'interviews. Ainsi, le consentement des intéressés, des personnes ressources, des responsables des réseaux et des leaders des associations identitaires, a permis que les centres de santé partenaires du programme national de lutte contre le sida et les IST et les ONG spécialisées en matière de santé des populations clés au niveau des régions des six villes couvertes par l'enquête puissent servir de sites de collecte des données aussi bien comportementales que biologiques. Dans chaque région sanitaire, un ou plusieurs centres désignés comme étant les plus connus et les plus fréquentés par les populations cibles ont été choisis (Tableau I).

**Tableau I:** Répartition des centre inclus selon les villes et les régions au Togo en 2022

Région	Population de la région	Ville	Centre	Population enquêtée
Grand Lomé	2188377	Lomé	AFAZ	PS
			Réseau Cupidon	HSH
Maritime	1 346615	Tsévié	Association EDV	HSH et PS
Plateaux	1 635946	Kpalimé	Association EDV	HSH et PS
Centrale	795529	Sokodé	Association EVT RC	HSH et PS
Kara	985512	Kara	AED	HSH
			ONG FAMME	PS
Savanes	1 143 520	Dapaong	ONG SAC Santé	HSH et PS
**Total**	**8 095 499**	**-**	**-**	**-**

* AFAZ (Association des femmes amazones zen); EDV (Association espoir de vivre); EVT RC (Association espoir vie Togo région centrale); AED (Association action espoir pour demain); FAMME (Force en action pour le mieux-être de la femme et de l'enfant); SAC Santé (Solidarité pour l'autopromotion communautaire en santé)

Pour être incluse comme HSH, la personne devait : i) être un homme biologique; ii) déclarer avoir eu au moins une fois un rapport sexuel anal (réceptif ou insertif) avec un homme au cours des six derniers mois; iii) résider habituellement dans la ville d'enquête depuis au moins trois mois au moment de l'enquête.

Pour être incluse comme PS, la personne devait : i) être une femme biologique. ii) avoir comme principale source de revenus l’échange de rapports sexuels contre de l'argent au cours des douze derniers mois; iii) résider habituellement dans la ville d'enquête avec une durée de séjour de plus de trois mois au moment de l'enquête.

**Figure 1:**
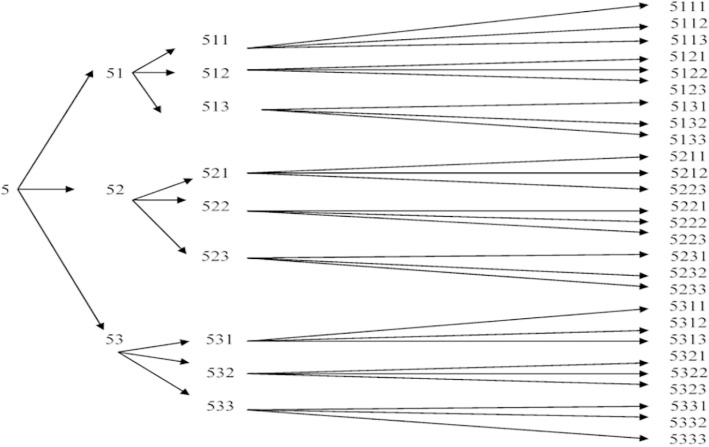
Principe de l’échantillonnage dirigé par les répondants pour le point de départ 5 pour les coupons

En plus de ces critères, chaque HSH ou PS devait avoir au moins 15 ans et avoir reçu un coupon de référence valable sauf pour les « graines », c'est-à-dire les tous premiers participants à l’étude. En effet, les coupons sont des petites fiches avec des numéros que les personnes incluses utilisent pour recruter d'autres personnes. La numérotation de ces coupons est particulière et permet d'avoir une idée du réseau de chaque individu. Chaque recruteur dispose d'un nombre moyen de trois coupons (Fig. 1).

### Taille de l’échantillon

La taille de l’échantillon a été déterminée à partir de la formule de proportion unique modifiée avec l'effet de conception recommandé par la méthodologie SMART *(Standardized monitoring and assessment of relief and transitions)* [18].


$$N=D\frac{Z_{\alpha }^{2}p(1-p)}{e^{2}}$$

N : taille de l’échantillon

Za : valeur de l’écart réduit pour un risque alpha de 5 % (Za : 1,96)

p : prévalence initiale de VIH. Selon la dernière SSG de 2017 [4], elle était de 22 % chez les HSH et 13 % chez les PS) e : 2 % (précision)

D : 2 % (effet plan)

En considérant 10 % de non-réponses, l’échantillon minimal pour notre étude était de 2 400 HSH et 3 650 PS en arrondissant à la cinquantaine supérieure.

### Recrutement des HSH et PS pour la collecte des données comportementales et de séroprévalence VIH/syphilis

La méthode d’échantillonnage basée sur les répondants (*Respondent-driving sampling* – RDS) a été utilisée pour le recrutement [2]. Les populations clés HSH et PS constituent dans la plupart des pays africains des populations fortement stigmatisées, organisées en réseaux, ce qui les rend difficiles d'accès à travers les méthodes conventionnelles de collecte de données. En réponse à cette situation, une méthode spécifique d’échantillonnage a été développée pour se rapprocher des méthodes d’échantillonnage probabiliste à travers un processus de référencement par les pairs pour ces populations difficiles d'accès. Cette approche se prête particulièrement bien au recrutement de populations cachées et difficiles d'accès [9]. Le RDS se distingue du mode de recrutement de type « boule de neige » usuel par certaines caractéristiques permettant d'obtenir un échantillonnage probabiliste représentatif des réseaux des populations cibles de l’étude, à condition que les hypothèses soient respectées.

Selon cette méthode, les chercheurs, avec leur capacité à recruter et à mobiliser les personnes de leurs réseaux pour participer à l’étude, choisissent de manière raisonnée les « graines », c'est à dire les tous premiers participants comme point de départ de la chaîne de recrutement. Après leur interview, ces premiers participants iront recruter d'autres personnes à enquêter. Cette première série d'enquêtés recrutés par les graines de l'enquête constitue ce qu'on appelle la première vague. Cette vague recrutera à son tour d'autres personnes qui vont constituer la deuxième vague de participants. Cette deuxième vague sera chargée de recruter la prochaine vague jusqu’à ce que la taille voulue de l’échantillon soit atteinte.

### Collecte des données

Les données comportementales ont été collectées à l'aide d'un entretien direct (face à face) par des enquêteurs formés issus des populations clés cibles. Les interviews ont été réalisées dans la langue (français, mina, kabyè, kotocoli, moba, éwé) couramment parlée par l'enquêté, et les réponses recueillies et saisies par l'enquêteur. L'enquête a été conduite dans les sites de l’étude, en privé, en garantissant la confidentialité des informations fournies par les répondants.

Les données biologiques ont été collectées par des techniciens de laboratoire formés dans les différents sites de l’étude. Après un conseil prétest, les prélèvements de sang ont été réalisés dans des tubes de prélèvement contenant de l'acide éthylène diamine tétra-acétique (EDTA) sous vide. Pour le VIH, le dépistage rapide sur site a été fait conformément à l'algorithme national de dépistage du VIH conforme à la stratégie II de l'OMS/ONUSIDA recommandant l'utilisation de deux résultats de test réactif consécutifs pour établir un diagnostic positif de VIH. Il a été utilisé en première intention le SD Bioline/HIV Syphilis Duo^®^, et ensuite un second test discriminant, en l'occurrence le First Response HIV 1.2.0^®^. La réalisation des tests pour la syphilis s'est faite dans un premier temps sur place à l'aide des tests de diagnostic rapide (HIV Syphilis Duo^®^) selon les recommandations de l'OMS, en vue d'une prise en charge rapide, et dans un deuxième temps, d'autres tests tréponémiques ont été réalisés au laboratoire de référence pour la confirmation ou le titrage des échantillons positifs au test rapide. Les services liés à la disponibilité des résultats biologiques, le *counseling post-test* et la référence vers des structures de soins et de traitements étaient offerts immédiatement après les tests rapides à tous les participants ayant accepté de se soumettre au dépistage.

### Traitement et analyses statistiques des données

Le traitement des données collectées s'est déroulé en six étapes principales : vérification journalière des questionnaires; saisie des données; saisie et vérification de la base de données de coupons; saisie et vérification de la base de données de laboratoire; apurement, et enfin tabulation. Les variables qualitatives ont été présentées sous forme de proportion.

Une analyse univariée (test de Chi^2^ d'indépendance) a d'abord été réalisée pour identifier les variables associées à l'infection du VIH. Le seuil de significativité a été d'abord fixé à 20 %. Après cette étape, les variables associées ont été introduites dans un modèle de régression logistique binaire pour une analyse multivariée, afin d'identifier des facteurs associés à l'infection au VIH. Le seuil de significativité était fixé à 5 %. Les estimations de poids ont été réalisées à l'aide de RDS Analyst^®^ (RDS-A), version 7.1.38 [7]. La version 16 du logiciel Stata^®^ (Stata Corporation, College Station, TX) a été utilisée pour calculer les statistiques descriptives et la modélisation.

### Aspects éthiques et réglementaires

L'avis écrit du Comité de bioéthique pour la recherche en santé du Togo a été obtenu avant la mise en œuvre de l'enquête (avis n° 009/2022/ CBRS du 24 mai 2022). Les personnes chargées de la mise en œuvre de cette enquête se sont conformées à toutes les procédures dudit comité. Chaque participant a donné son consentement libre, éclairé et écrit. La collecte des données s'est déroulée dans le respect de la confidentialité et de l'anonymat des données collectées, de la dignité et de la liberté de chaque individu invité à y participer et libre de se retirer à tout moment de l'enquête.

## Résultats

### Données comportementales et de séroprévalence

#### Caractéristiques socio-démographiques

Au total, 2 110 HSH et 3 221 PS ont été inclus dans notre étude. Les sujets âgés de 15 à 24 ans représentaient 53,3 % des HSH et 49,7 % des PS. La moitié des PS et HSH avait atteint le niveau d'instruction secondaire. En ce qui concerne la situation matrimoniale, la majorité des HSH (91,7 %) a déclaré ne s’être jamais mariée. C’était aussi le cas chez les PS dans 78,5 % des cas (Tableau II).

**Tableau II:** Caractéristiques socio-démographiques des HSH et PS enquêtés au Togo en 2022

	HSH (n= 2 110)	PS (%) (n= 3 221)
	n	%	n	%
Groupe d’âge				
15-24 ans	1 125	53,3	1 598	49,6
25 ans et plus	985	46,7	1 623	50,4
Situation matrimoniale				
jamais marié(e)	1 935	91,7	2 528	78,5
actuellement marié(e)	112	5,3	171	5,3
divorcé(e)/séparé(e)/veuf(ve)	63	3,0	522	16,2
Niveau d'instruction				
non scolarisé(e)	38	1,8	290	9,0
primaire	120	5,7	824	25,6
secondaire	975	46,2	1729	53,7
supérieur	964	45,7	367	11,4
école coranique	13	0,6	11	0,3

#### Orientations et comportements sexuels

Plus de la moitié (58,5 %) des HSH étaient homosexuels stricts, 41,5 % étaient bisexuels. Le rôle sexuel préférentiel était insertif dans 54,8 % des cas, réceptif dans 30,4 % des cas et versatile (l'un ou l'autre) dans 14,8 % des cas. Le nombre médian de partenaires sexuels était de 2 au cours des six derniers mois. Deux tiers (65 %) des HSH ont rapporté avoir utilisé un préservatif lors de leur dernier rapport sexuel anal avec un autre partenaire masculin. Parmi les 731 HSH (34,6 %) ayant eu un rapport sexuel avec une femme (anal et/ou vaginal) lors des six derniers mois, 32,6 % ont rapporté ne pas avoir utilisé de préservatif. En ce qui concerne le lubrifiant à base d'eau, 76,9 % des HSH ont signalé toujours l'utiliser, 22,6 % ne l'utilisaient que quelquefois et 0,4 % ont rapporté ne l'avoir jamais utilisé. L'utilisation toujours concomitante du préservatif et du lubrifiant à eau a été rapportée chez 68,1 %, alors que 31,9 % ont rapporté ne pas toujours utiliser les deux ensemble.

Dans 56,4 % des cas, les PS ont déclaré avoir eu au moins trois clients payants parmi leurs partenaires sexuels dans les sept derniers jours. Plus de la moitié des PS (51,8 %) a déclaré avoir toujours utilisé le préservatif avec les partenaires sexuels payants et 37,3 % ont déclaré l'avoir toujours utilisé avec les partenaires sexuels non payants au cours des sept derniers jours. Sept PS sur dix (71,7 %) ont déclaré avoir utilisé le préservatif lors du dernier rapport sexuel avec un client payant. Parmi les PS qui avaient rapporté n'avoir pas utilisé un préservatif lors du dernier rapport sexuel, 56,9 % avaient évoqué le refus du partenaire sexuel et 17,3 % le fait de se voir proposer plus d'argent pour un rapport sans protection.

### Prévalence du VIH, de la syphilis et des facteurs associés au VIH (prévalences pondérées avec IC 95 %)

#### Hommes ayant des rapports sexuels avec d'autres hommes

Dans notre étude, la prévalence pondérée du VIH chez les HSH était estimée à 8,7 % (IC 95 %; 7,5 % à 9,9 %). La région ayant la prévalence pondérée la plus élevée était Lomé avec 30,2 % (25,2 %-35,1 %) (Tableau III).

**Tableau III:** Prévalence pondérée du VIH chez les HSH et PS enquêtés au Togo en 2022 (IC 95 %)

Région	Testés	Cas positifs	Prévalence pondérée VIH (%)
		**HSH**	
**Grand Lomé**	314	100	30,2 (25,2-35,1)
**Tsévié**	459	24	4,6 (2,9-6,3)
**Kpalimé**	336	7	1,9 (0,5-3,2)
**Sokodé**	199	11	5,5 (2,7-8,3)
**Kara**	524	15	2,8 (1,1-4,4)
**Dapaong**	251	6	2,2 (0,5-3,9)
**Total**	**2 083**	**163**	**8,7 (7,5-9,9)**
		**PS**	
**Grand Lomé**	622	51	7,1 (5,1-9,1)
**Tsévié**	451	61	13,2 (10,1-16,3)
**Kpalimé**	687	18	2,2 (1,1-3,3)
**Sokodé**	328	24	6,9 (4,1-9,6)
**Kara**	523	45	7,4 (5,1-9,6)
**Dapaong**	589	25	3,8 (2,3-5,3)
**Total**	**3 200**	**224**	**5,8 (5-6,6)**

La prévalence du VIH chez les HSH de 25 ans et plus était de 10,5 % (9,5 % à 11,5 %) et de 5,5 % (4,6 % à 6,4 %) chez les 15 à 24 ans. En analyse multivariée, les HSH qui seraient le plus à risque de contracter le VIH sont ceux ayant :

- un âge supérieur à 25 ans (rapport de cotes OR = 1,71; 1,49-1,93; p = 10^-8^)

- une notion de déchirure de préservatif au cours des douze derniers mois (OR = 1,26; 1,06-1,46; p = 0,001)

- eu plus de deux partenaires sexuels masculins au cours des six derniers mois (OR = 1,96; 1,54-2,38; p = 10^-7^)

Seraient moins exposés au risque de contracter le VIH les HSH :

- qui ont fait l'usage systématique concomitant du préservatif avec des lubrifiants au cours des six derniers mois (OR = 0,68; 0,52- 0,84; p = 10^-8^)

- qui ont eu systématiquement recours au préservatif avec des partenaires réguliers au cours des six derniers mois (OR = 0,74; 0,69-0,79; p =10^-6^)

- qui ont une bonne connaissance sur le VIH (OR = 0,66; 0,59-0,73; p = 10^-7^).

Pour ce qui est de la syphilis, 12 cas positifs ont été retrouvés chez les 2 083 HSH testés, soit une prévalence pondérée de 0,6 % (0,3 %-0,9 %).

#### Professionnelles du sexe

Dans l'ensemble, la prévalence pondérée du VIH chez les PS au Togo est estimée à 5,8 % (5 %-6,6 %) (Tableau III). En analyse multivariée, les PS ayant présenté au moins un signe d'IST (liquide anormal, plaies ou boutons sur le sexe) au cours des six derniers mois sont plus à risque de contracter le VIH que celles n'ayant aucun signe d'IST (OR=1,24; 1,19-1,29; p=10^-8^). Les PS ayant une bonne connaissance sur le VIH sont moins à risque de contracter le VIH (OR = 0,91; 0,85-0,97; p = 0,05). S'agissant de l'ancienneté dans la profession, les PS ayant cumulé au moins trois années dans la profession sont plus à risque de contracter le VIH (OR = 3,76; 3,45-4,07; p = 10^-9^). La prévalence pondérée de la syphilis était de 0,2 % chez les PS au Togo.

## Discussion

Notre étude a permis de déterminer la prévalence du VIH et de la syphilis dans les populations HSH et PS du Togo en 2022. Les données existantes étaient vieilles de 5 ans et il était indispensable de les actualiser afin de mieux orienter les mesures de prévention et de prise en charge adaptées à ces populations. Ainsi, en 2022, la prévalence pondérée du VIH au Togo était de 8,7 % (7,5 %-9,6 %) chez les HSH et de 5,8 % (5 %-6,6 %) dans les populations de PS. Quant à la syphilis, sa prévalence pondérée était de 0,2 % (0,3 %-0,9 %) chez les HSH et de 0,6 % (0 %-0,3 %) chez les PS. L'une des principales forces de notre étude est la méthode d’échantillonnage mise en place. En effet, la méthode RDS nous a permis d'atteindre une grande partie des populations cibles, mais aussi d'avoir des échantillons assez représentatifs. Cependant, notre étude présente certaines limites :

- elle n'a pas pu être réalisée dans toutes les villes du pays pour des raisons financières. Cela explique d'ailleurs le fait que nous n'ayons pas eu les deux effectifs nécessaires calculés pour notre étude, affaiblissant ainsi dans une certaine mesure la validité de nos résultats;

- la méthode de collecte des données comportementales (interview face à face) pourrait avoir engendré un biais d'information et un biais lié à l'enquêteur. En effet, les enquêtés pourraient volontairement taire certaines de leurs habitudes sexuelles par peur de jugement.

La prévalence du VIH dans notre étude chez les HSH et chez les PS est respectivement 5 fois et 3 fois supérieure à la prévalence nationale du VIH au Togo estimée à 1,7 % en 2022. Nous notons une baisse de la prévalence dans ces populations clés par rapport à celle de 2011 (date de démarrage dans le pays de la mise en place des programmes de prévention adaptés aux HSH et aux PS avec l'offre de paquets complets de services), qui était de 19 % et de 13 % respectivement chez les HSH et les PS [5, 19]. Cette baisse documente l'effet de l'extension de l'offre de services adaptés mise en place dans le pays depuis 10 ans, avec l'appui des partenaires techniques et financiers (comme le Fonds mondial de lutte contre le sida, la tuberculose et le paludisme, et *l'U.S. Agency for International Development,* USAID. En effet, avant 2011, il n'existait pas dans le pays de programme national de prévention structuré destiné à ces deux populations vulnérables.

La prévalence de la syphilis est faible dans le pays aussi bien chez les HSH (0,6 %, 0,3 %-0,9 %) que parmi les PS (0,2 %, 0 %-0,3 %). Elle est comparable à celle des femmes enceintes en consultation prénatale : prévalence stable depuis une dizaine d'années [1]. Cependant, l'incidence du VIH serait deux fois plus élevée chez les HSH ayant la syphilis [21], documentant ainsi que les comportements à risque favorisent cette double infection.

En ce qui concerne les déterminants de l'infection au VIH, la majorité des études dans la littérature a trouvé quasiment les mêmes facteurs associés au VIH chez les HSH et les PS que ceux identifiés dans la nôtre [3, 5, 14, 19]. Les facteurs constatés dans notre étude comme étant associés à moins de risques d'avoir le VIH sont intimement liés à l'accès des populations clés aux paquets de service mis en place pour la prévention du VIH. L'environnement juridique et socioculturel qui n'est pas toujours favorable aux HSH et PS dans le pays, comme dans la majorité des pays en Afrique subsaharienne, constitue un obstacle à l'accès aux soins. Des études en Afrique sub-saharienne ont montré que la prévalence du VIH était significativement plus élevée dans les pays où il existait des mesures punitives et des lois criminalisant l'homosexualité [12] et la prostitution [11]. C'est pour lutter contre ces problèmes que la stratégie globale de l'ONUSIDA comporte un axe de lutte contre la stigmatisation et la discrimination.

## Conclusion

Notre étude a permis de noter une tendance à la baisse de la prévalence du VIH chez les HSH et les PS sur une période de 10 ans. Malgré cette tendance, la prévalence du VIH dans ce groupe de population reste encore élevée par rapport à celle de la population générale, au regard des objectifs visés par le programme national de lutte contre le VIH et les IST.

Même si la mise en place de nouveaux moyens de prévention et de prise en charge du VIH comme la PrEP *(Pre-exposure prophylaxis)* et l'autotest devrait permettre d'améliorer le profil épidémiologique de cette affection dans les populations clés, il est important de continuer la mise en œuvre des interventions dynamiques, innovantes et adaptées à ces populations et de lever certains obstacles à l'accès aux soins comme les problèmes de stigmatisation.

## Remerciements

Les auteurs remercient le ministère de la Santé, de l'hygiène publique et de l'accès universel, le Programme national de lutte contre le sida, les hépatites et les IST et le Conseil national de lutte contre le VIH/sida (CNLS) qui ont proposé cette étude dans le cadre de la mise en œuvre d'un plan stratégique national 2020-2025.

## Financement

Cette étude a bénéficié du financement du Fonds mondial de lutte contre le sida, la tuberculose et le paludisme au Togo.

## Contribution des auteurs

Conception de l’étude : Palokinam PITCHE, Anoumou DAGNRA, Panawé KASSANG

Collecte et analyse des données : Nadjombé GBANDI, Kodzo DEKU, Charles LIMAZIE

Rédaction du manuscrit : Panawé KASSANG, Sefako AKAKPO

Supervision et correction du manuscrit : Palokinam PITCHE, Anoumou DAGNRA

## Conflits d'intérêts

Les auteurs ne déclarent aucun conflit d'intérêt.
