# Supplementary material for: Prevalence of HIV, syphilis and factors associated with HIV infection among men who have sex with men and among female sex workers in Togo in 2022
Source: Med Trop Sante Int. 2024 Jul 8;4(3):mtsi.v4i3.2024.433. [Article in French] doi: 10.48327/mtsi.v4i3.2024.433 (PMC11811740; doi:10.48327/mtsi.v4i3.2024.433)
Supplement: Related texts

# Research article: Prevalence of HIV, syphilis and factors associated with HIV infection among men who have sex with men and among female sex workers in Togo in 2022

## Introduction

In 2022, nearly 36 million people worldwide were living with HIV, according to UNAIDS. Global HIV prevalence among adults (aged 15-49) was 0.7%, but was much higher among key populations: female sex workers (FSWs) (2.5%); men who have sex with men (MSM) (7.7%); injecting drug users (IDUs) (5%); transgender people (10.3%); and prisoners (1.4%) [15]. About 110,000 (66,000 to 190,000) new HIV infections were reported in Central and West Africa in the same year [15]. It has been reported that prevalence rates are very high in countries such as Cameroon (34%) [17], Nigeria (34%) [20] and the Central African Republic (41%) [13]. Despite the vastly improved availability of HIV prevention and care in resource-limited countries in sub-Saharan Africa, certain key populations such as FSWs and MSM continue to be poorly reached by these service packages [10], due to stigma and discrimination [6]. One of the obstacles to achieving the objectives of controlling HIV infection in these populations is the problem of stigmatization. This situation has led some countries, such as Togo, to set up appropriate services to make HIV interventions more accessible to these populations [8]. Since 2011, Togo has implemented a prevention program tailored to key populations (MSM, FSWs, IDU) and a system of epidemiological surveillance of HIV through second-generation surveillance surveys (SSG) in these key populations. This enables the measurement of the impact of interventions [16]. The objective of this study is to ascertain the prevalence of HIV and syphilis and the factors associated with HIV among MSM and FSWs in Togo in 2022.

## Materials and methods

### Study design and period

This descriptive and analytical cross-sectional observational study was conducted among MSM and FSWs from August 30, 2022, to September 30, 2022, in the six health regions of Togo.

### Pre-study preparation

Prior to the survey, an exploratory and initial consultation mission was conducted in the country's six health regions with the objective of gaining a more comprehensive understanding of the context in which the survey would be implemented. The survey was conducted between June 28 and July 4, 2022. The primary objective of the mission was to gather data and information that would facilitate the final adjustments to the operational strategy for implementing the survey. Furthermore, the mission provided an opportunity to secure the support and commitment of the target population and stakeholders, to discuss the objectives and sampling method, and to establish connections with the MSM community, the FSWs, and the various partners.

### Selection of sites and study population

The preliminary investigation and preliminary consultation led to closer contact with the MSM and FSW communities, and to the participatory and inclusive involvement of their members in identifying the interview sites. With the consent of the relevant individuals, resource persons, network managers, and leaders of identity associations, the partner health centers of the national AIDS and STI program and NGOs specializing in the health of key populations in the regions of the six towns covered by the survey were utilized as sites for the collection of both behavioral and biological data. In each health region, one or more centers were selected as the most well-known and most frequently visited by the target populations (Table I).

**Table I:** Distribution of included centers by city and region in Togo in 2022

Region	Population of region	City	Center	Population surveyed
Grand Lomé	2188377	Lomé	AFAZ	FSWs
			Réseau Cupidon	MSM
Maritime	1 346615	Tsévié	Association EDV	MSM & FSWs
Plateaux	1 635946	Kpalimé	Association EDV	MSM & FSWs
Centrale	795529	Sokodé	Association EVT RC	MSM & FSWs
Kara	985512	Kara	AED	MSM
			ONG FAMME	FSWs
Savanes	1 143 520	Dapaong	ONG SAC Santé	MSM & FSWs
**Total**	**8 095 499**	**-**	**-**	**-**

* AFAZ (Association des femmes amazones zen); EDV (Association espoir de vivre); EVT RC (Association espoir vie Togo région centrale); AED (Association action espoir pour demain); FAMME (Force en action pour le mieux-être de la femme et de l'enfant); SAC Santé (Solidarité pour l'autopromotion communautaire en santé)

To be included as an MSM, the individual in question had to fulfill three criteria: (i) be a biological male, (ii) report having had anal sex (receptive or insertive) with a man at least once in the past six months, and (iii) have been ordinarily resident in the survey city for at least three months at the time of the survey.

To be included as an FSW, the individual had to meet the following criteria: (i) be a biological female; (ii) have exchanged sex for money as a primary source of income in the past twelve months; (iii) be a resident of the survey city for a minimum of three months at the time of the survey.

In addition to these criteria, each MSM or FSW had to be at least 15 years of age and have received a valid reference coupon, with the exception of the “seeds,” or the very first participants in the study. The coupons are small cards bearing numbers, which the participants use to recruit others. The numbering of these coupons is unique and provides insight into the size and structure of each individual's network. The average recruiter possesses approximately three coupons (Fig. 1).

**Figure 1:** Principle of respondent-driven sampling for starting point 5 for coupons

### Sample size

The sample size was calculated using the single proportion formula, modified with the design effect recommended by the SMART methodology (Standardized Monitoring and Assessment of Relief and Transitions) [18].


$$N=D\frac{Z_{\alpha }^{2}p(1-p)}{e^{2}}$$

N: sample size

Zα: value of the reduced error for an alpha risk of 5% (Zα: 1.96)

p: baseline HIV prevalence. According to the last SSG in 2017 [4], it was 22% in MSM and 13% in FSWs)

e: 2% (accuracy)

D: 2% (design effect)

In consideration of the 10% non-responses, the minimum sample size for our study was 2,400 MSM and 3,650 FSWs, rounded up to the nearest 50.

### Recruitment of MSM and FSWs to collect behavioral and HIV/syphilis seroprevalence data

The Respondent-Driven Sampling (RDS) method was employed for the purpose of recruitment [2]. In most African countries, the key populations of MSM and FSWs are highly stigmatized and organized in networks, which makes them difficult to access using conventional data collection methods. In response to this situation, a specific sampling method has been developed to approximate probability sampling methods through a peer referral process for these populations that are difficult to reach. This approach is particularly well-suited to the recruitment of hidden and hard-to-reach populations [9]. The RDS differs from the conventional “snowball” approach to recruitment in that it enables the acquisition of a probability sample that is representative of the networks of the study's target populations, provided that the underlying assumptions are met. In this approach, researchers leverage their ability to recruit and mobilize individuals from their networks to participate in the study, selecting the initial participants, or “seeds,” as the foundation for the recruitment chain. Subsequently, the initial participants will proceed to recruit additional individuals for interviews. The initial series of respondents recruited by the survey's initial participants constitutes what is known as the first wave. Subsequently, this initial cohort of participants will themselves recruit other individuals, thereby constituting the second wave of respondents. The second wave will be responsible for recruiting the subsequent wave until the desired sample size is reached.

### Data collection

Behavioral data was collected via direct (face-toface) interviews conducted by trained interviewers from the key target populations. The interviews were conducted in the language most commonly spoken by the respondents, and the answers were collected and entered by the interviewers. The survey was conducted in the study sites in private, thus ensuring the confidentiality of the information provided by the respondents. Biological data were collected by trained laboratory technicians at the various study sites. Following pre-test counseling, blood samples were collected in EDTA-containing vacuum tubes. For HIV, rapid on-site screening was conducted in accordance with the national HIV screening algorithm, in alignment with the World Health Organization (WHO) and the Joint United Nations Programme on HIV/AIDS (UNAIDS) strategy II, which recommends the use of two consecutive reactive test results to establish a positive HIV diagnosis. The initial diagnostic test employed was the SD Bioline/HIV Syphilis Duo^®^, followed by a second, more discriminatory test, the First Response HIV 1.2.0^®^. Syphilis tests were initially conducted onsite using rapid diagnostic tests (HIV Syphilis Duo^®^) in accordance with WHO recommendations, with the objective of rapid management. Subsequently, other treponemal tests were carried out at the reference laboratory for confirmation or titration of samples that were positive to the rapid test.

Immediately following the administration of the rapid tests, participants who consented to screening were offered a range of services related to the availability of biological results, post-test counseling, and referral to care and treatment facilities.

### Data processing and statistical analysis

The data were subjected to six principal processing stages, namely daily examination of the questionnaires, data entry, entry and verification of the voucher database, entry and verification of the laboratory database, reconciliation, and finally tabulation. Qualitative variables were presented in the form of proportions.

A univariate analysis (chi-square test of independence) was initially conducted to identify the variables associated with HIV infection. The initial significance threshold was set at 20%. Subsequently, the variables identified as being associated with HIV infection were entered into a binary logistic regression model for multivariate analysis. This was done in order to identify the factors associated with HIV infection. The significance level was set at 5%. Weight estimates were generated using RDS Analyst^®^ (RDS-A), version 7.1.38 [7]. The Stata^®^ software version 16 (Stata Corporation, College Station, TX) was employed to compute descriptive statistics and to perform modeling.

### Ethical and regulatory aspects

The written opinion of Togo's Bioethics Committee for Health Research was obtained prior to the survey being conducted (opinion no. 009/2022/ CBRS of 24 May 2022). Those responsible for implementing the survey ensured that all procedures set forth by the aforementioned committee were adhered to. Each participant provided informed consent in writing. The data collection process was conducted in accordance with the principles of confidentiality and anonymity, as well as the dignity and freedom of each individual invited to participate in the survey. Participants were informed of their right to withdraw from the survey at any time.

## Results

### Behavioral and seroprevalence data

#### Socio-demographic characteristics

A total of 2,110 MSM and 3,221 FSWs were included in the study. The subjects were distributed as follows: 53.3% of the MSM and 49.7% of the FSWs were between the ages of 15 and 24. Half of the FSWs and MSM had completed secondary education. With regard to marital status, the majority of MSM (91.7%) indicated that they had never been married. This was also the case for 78.5% of FSWs (Table II).

**Table II:** Socio-demographic characteristics of observed MSM and FSW in Togo in 2022

	MSM (n= 2 110)	FSW (%) (n= 3 221)
	n	%	n	%
Age group				
15-24 years	1 125	53,3	1 598	49,6
25 years and over	985	46,7	1 623	50,4
Marital status				
never married	1 935	91,7	2 528	78,5
currently married	112	5,3	171	5,3
divorced/separated/widowed	63	3,0	522	16,2
Level of education				
no education	38	1,8	290	9,0
primary	120	5,7	824	25,6
secondary	975	46,2	1729	53,7
higher	964	45,7	367	11,4
Koranic school	13	0,6	11	0,3

#### Sexual orientation and behavior

A total of 58.5% of the MSM were exclusively homosexual, while 41.5% were bisexual. The majority of respondents (54.8%) identified as insertive, while 30.4% identified as receptive and 14.8% identified as versatile (having one or the other role). The median number of sexual partners in the previous six months was two. Two-thirds (65%) of the MSM reported having used a condom during their most recent anal intercourse with another male partner. Among the 731 MSM who had engaged in sexual intercourse with a woman (anal and/or vaginal) in the previous six months, 32.6% reported that they had not used a condom. With regard to the use of water-based lubricant, 76.9% of MSM reported consistent use, 22.6% reported occasional use, and 0.4% reported no use. The proportion of individuals who reported always using condoms and aqueous lubricant together was 68.1%, while 31.9% reported not always using both together.

In 56.4% of cases, FSWs reported having had at least three paying clients among their sexual partners in the previous seven days. A greater proportion of FSWs (51.8%) indicated that they had consistently utilized condoms with their paying sex partners, while 37.3% reported the same with their non-paying sex partners over the past seven days. A majority of FSWs (71.7%) indicated that they had used a condom during their most recent sexual encounter with a paying client. Among the FSW respondents who indicated that they had not used a condom during their most recent sexual encounter, 56.9% reported that their sexual partner had refused to use a condom, while 17.3% stated that they had been offered more money for unprotected sex.

### Prevalence of HIV, syphilis and factors associated with HIV (weighted prevalences with 95% CI)

#### Men who have sex with men

In our study, the weighted prevalence of HIV among men who have sex with men (MSM) was estimated at 8.7% (95% CI: 7.5% to 9.9%). The region with the highest weighted prevalence was Lomé, with a prevalence of 30.2% (95% CI: 25.2% to 35.1%) (Table III).

**Table III:** Weighted HIV prevalence among observed MSM and FSW in Togo in 2022 (95 % CI)

Region	Tested	Positive cases	Weighted HIV prevalence (%)
		**HSH**	
**Grand Lomé**	314	100	30,2 (25,2-35,1)
**Tsévié**	459	24	4,6 (2,9-6,3)
**Kpalimé**	336	7	1,9 (0,5-3,2)
**Sokodé**	199	11	5,5 (2,7-8,3)
**Kara**	524	15	2,8 (1,1-4,4)
**Dapaong**	251	6	2,2 (0,5-3,9)
**Total**	**2 083**	**163**	**8,7 (7,5-9,9)**
		**PS**	
**Grand Lomé**	622	51	7,1 (5,1-9,1)
**Tsévié**	451	61	13,2 (10,1-16,3)
**Kpalimé**	687	18	2,2 (1,1-3,3)
**Sokodé**	328	24	6,9 (4,1-9,6)
**Kara**	523	45	7,4 (5,1-9,6)
**Dapaong**	589	25	3,8 (2,3-5,3)
**Total**	**3 200**	**224**	**5,8 (5-6,6)**

HIV prevalence among MSM aged 25 and over was 10.5% (9.5% to 11.5%) and 5.5% (4.6% to 6.4%) among those aged 15 to 24. In a multivariate analysis, the MSM most at risk of contracting HIV were those with:

- age over 25 (OR = 1.71; 1.49-1.93; p = 10^-8^)

- a history of condom tears in the last twelve months (OR = 1.26; 1.06-1.46; p = 0.001)

- more than two male sexual partners in the last six months (OR = 1.96; 1.54-2.38; p = 10^-7^)

MSM were less at risk of contracting HIV in case of:

- systematic use of condoms with lubricants in the last six months (OR = 0.68; 0.52- 0.84; p = 10^-8^)

- systematic use of condoms with regular partners in the last six months (OR = 0.74; 0.69-0.79; p =10^-6^)

- good knowledge of HIV (OR = 0.66; 0.59-0.73; p = 10^-7^)

As for syphilis, 12 positive cases were found among the 2 083 MSM tested, giving a weighted prevalence of 0.6% (0.3%-0.9%).

#### Women sex workers

The weighted prevalence of HIV among FSWs in Togo is estimated to 5.8% (5%-6.6%) (Table III). In a multivariate analysis, FSWs with at least one sign of STI (abnormal fluid, sores or pimples on the sex) in the last six months were found to be at greater risk of contracting HIV than those with no sign of STI (OR=1.24; 1.19-1.29; p=10-8). FSWs with a comprehensive understanding of HIV were less susceptible to contracting the virus (OR=0.91; 0.85-0.97; p=0.05). In terms of the length of time spent in the profession, FSWs with at least three years of experience were found to be at greater risk of contracting HIV (OR = 3.76; 3.45-4.07; p = 10^-9^).

The weighted prevalence of syphilis among FSWs in Togo was 0.2%.

## Discussion

The objective of this study was to determine the prevalence of HIV and syphilis in the MSM and FSW populations of Togo in 2022. The existing data were five years old and thus required updating in order to provide more accurate guidance for the development of prevention and care measures tailored to these populations. Consequently, in 2022, the weighted prevalence of HIV in Togo was 8.7% (7.5%-9.6%) among MSM and 5.8% (5%-6.6%) among FSWs. With regard to syphilis, the weighted prevalence was 0.6% (0.3%-0.9%) among MSM and 0.2% (0%-0.3%) among FSWs. One of the principal strengths of this study is the sampling method employed. The RDS method enabled us to reach a large proportion of the target populations and to obtain fairly representative samples. Nevertheless, it should be noted that our study is not without certain limitations:

- it was not feasible to conduct the study in every region in the country due to financial constraints. This explains why the requisite two numbers were not obtained for our study, thereby compromising the reliability of our findings to some extent;

- the method of collecting behavioral data (face-to-face interview) could have introduced an information bias and an interviewer bias. It is possible that respondents may have been reluctant to disclose certain aspects of their sexual habits, due to concerns about being judged.

In our study, the prevalence of HIV among MSM and FSWSs was found to be 5 times and 3 times higher, respectively, than the national HIV prevalence in Togo, which was estimated at 1.7% in 2022. A notable decline in prevalence has been observed in these key populations compared to 2011, the year in which the country initiated the implementation of targeted prevention programs for MSM and NSPs, accompanied by the provision of comprehensive service packages. In 2011, the prevalence among MSM and NSPs was estimated to be 19% and 13%, respectively (5,19). This decline can be attributed to the implementation of adapted services over the past decade, with the support of technical and financial partners, including the Global Fund to Fight AIDS, Tuberculosis and Malaria, and the US Agency for International Development (USAID). Prior to 2011, there was no structured national prevention program for these two vulnerable populations.

The prevalence of syphilis is low in the country, both among MSM (0.6%, 0.3%-0.9%) and FSWs (0.2%, 0%-0.3%). It is comparable to the prevalence among pregnant women attending antenatal clinics, which has been stable for around ten years [1]. However, the incidence of HIV is twice as high among MSM with syphilis [21], showing that high-risk behavior favors this dual infection. With regard to the determinants of HIV infection, the majority of studies in the literature have identified a similar set of factors associated with HIV in MSM and PS as those identified in our study [3, 5, 14, 19]. The factors found to be associated with a lower risk of HIV infection are closely linked to the access of key populations to the service packages put in place for HIV prevention. The legal and socio-cultural environment, which is not always conducive to the well-being of MSM and PS in the country, as in the majority of countries in sub-Saharan Africa, presents a challenge to accessing care. Studies conducted in sub-Saharan Africa have demonstrated that the prevalence of HIV is significantly higher in countries where there are punitive measures and laws that criminalize homosexuality and prostitution. To address these challenges, the UNAIDS global strategy prioritizes the elimination of stigma and discrimination.

## Conclusion

Our study demonstrated a downward trend in HIV prevalence among MSM and PS over a 10-year period. Notwithstanding this downward trend, HIV prevalence in this population group remains elevated in comparison to the general population, given the objectives of the national program to combat HIV and STIs.

Although the introduction of new HIV prevention and care methods, such as PrEP (pre-exposure prophylaxis) and self-testing, should help to improve the epidemiological profile of this condition in key populations, it is important to continue implementing dynamic, innovative interventions tailored to these populations and to remove certain obstacles to access to care, such as problems of stigmatization.

## Acknowledgements

The authors would like to thank the Ministry of Health, Public Hygiene and Universal Access, the National AIDS, Hepatitis and STI Control Programme and the National HIV/AIDS Control Council (CNLS) for proposing this study as part of the implementation of a national strategic plan for 2020-2025.

## Financing

The study was financed by the Global Fund to Fight AIDS, Tuberculosis and Malaria in Togo.

## Authors’ contributions

Study design : Palokinam PITCHE, Anoumou DAGNRA, Panawé KASSANG

Data collection and analysis: Nadjombé GBANDI, Kodzo DEKU, Charles LIMAZIE

Rédaction du manuscrit: Panawé KASSANG, Sefako AKAKPO

Supervision and correction of manuscript: Palokinam PITCHE, Anoumou DAGNRA

## Conflicts of interest

The authors declare no conflict of interest.
